# Endocuff‐Assisted Colonoscopy for Identifying Sessile Serrated Polyps and Adenomas During Routine Colorectal Cancer Screening: A Retrospective Cohort Study

**DOI:** 10.1002/jgh3.70173

**Published:** 2025-05-14

**Authors:** Ammad Javaid Chaudhary, Muhammad Saad Faisal, Abdullah Sohail, Hope Baldwin, Kevin Harris, Muhammad Shahzil, Muhammad Salman Faisal, Avi Toiv, Keith Mullins, Suraj Suresh

**Affiliations:** ^1^ Department of Internal Medicine Henry Ford Hospital Detroit Michigan USA; ^2^ Department of Internal Medicine University of Iowa Hospitals and Clinics Iowa City Iowa USA; ^3^ School of Medicine Wayne State University Detroit Michigan USA; ^4^ Department of Gastroenterology Henry Ford Hospital Detroit Michigan USA; ^5^ Department of Internal Medicine Weiss Memorial Hospital Chicago Illinois USA

**Keywords:** endocuff‐assisted colonoscopy, screening colonoscopy, sessile serrated polyps, tubular adenomas

## Abstract

**Background and Aims:**

Polyps located in less accessible areas of the colon, such as inner curves of flexures, are often difficult to visualize. Colonoscope attachments such as the Endocuff have been developed to improve the visualization of these polyps. We aimed to assess the utility of Endocuff‐assisted colonoscopy (EAC) in the detection of tubular adenomas and sessile serrated polyps (SSP) compared to conventional colonoscopy during routine colorectal cancer screening.

**Patients and Methods:**

This retrospective cohort study included patients who underwent colorectal cancer screening with either conventional colonoscopy or EAC between November 2022 and March 2023. The primary outcomes were SSP and tubular adenoma detection rates. Secondary outcomes included total procedure time, cecal intubation time, and ileal intubation rates.

**Results:**

Of the 435 patients included, 189 (43%) underwent EAC, and 246 (57%) underwent conventional colonoscopy. The mean ± standard deviation number of polyps detected was 1.7 ± 2.2, the mean procedure time was 18.7 ± 7.5 min, and the mean cecal intubation time was 4.4 ± 3.3 min, with no significant differences between groups. A smaller proportion of patients in the EAC group had successful ileal intubation (14% vs. 55%; *p* < 0.01). The tubular adenoma detection rate was similar between EAC and conventional colonoscopy (41% vs. 39%; *p* = 0.70), but the SSP detection rate was significantly higher with EAC (16% vs. 8.5%; *p* < 0.01).

**Conclusion:**

EAC may enhance the detection of difficult‐to‐visualize SSPs during screening colonoscopies without affecting overall procedure time. However, physicians should consider the examination indication when selecting EAC, as ileal intubation may be more challenging.

## 1 Introduction

Colorectal cancer (CRC) is the third most common cancer in the United States and worldwide [1]. The incidence and mortality of CRC have decreased due to regular screening practices that promote early detection, prompt removal of polyps, and timely treatment initiation [2]. Various screening tests for CRC are available, including direct visualization with sigmoidoscopy or colonoscopy, computed tomography (CT) colonography, fecal immunochemical testing, and multi‐target stool DNA testing [3]. Among these, colonoscopy facilitates direct visualization and removal of polyps and is the most sensitive and specific test for CRC screening [4].

However, colonoscopy presents clinical challenges. The effectiveness of CRC screening with colonoscopy relies on the direct visualization of polyps, pre‐cancerous lesions, and colorectal carcinomas. This method can be inadequate for identifying polyps in less accessible areas of the colon, such as behind folds and inner curves of flexures. Polyps, especially flat ones like sessile serrated polyps (SSPs), are often missed in these locations. SSPs are harder to visualize and tend to be located in the right‐sided colon. Additionally, incomplete bowel preparation and challenging anatomical configurations can complicate the visualization of the right‐sided colon, leading to colonoscope looping [5].

To address these limitations, various attachments have been developed to enhance visualization during colonoscopy, including Endo Rings and the Endocuff or Endocuff Vision (Olympus, USA) [6]. The Endocuff is a distal cap attachment for colonoscopes, featuring soft projections that flatten during insertion and extend outward during withdrawal to improve examination of colonic folds. Approved by the US Food and Drug Administration (FDA) in 2012, the Endocuff was designed to prevent scope slippage, aid in tip stabilization, and enhance inspection of lesions behind mucosal folds [7].

Various studies have compared endocuff–assisted colonoscopy (EAC) to conventional colonoscopy with positive results favoring EAC, and some studies have shown increased adenoma detection rates with the EAC [8]. However, whether EAC is advantageous for visualizing SSPs, which are flatter and more difficult to identify in the general population of individuals undergoing screening colonoscopies, has not been rigorously investigated. Therefore, we conducted a retrospective cohort study to evaluate the utility of EAC versus conventional colonoscopy in identifying SSPs and tubular adenomas during routine CRC screening and surveillance.

## 2 Methods

### 2.1 Patient Selection and Data Collection

We conducted a retrospective cohort study involving adults aged 18 years and older who underwent screening or surveillance colonoscopy at one of Henry Ford Hospital's five ambulatory surgical centers between November 2022 and March 2023. Data collection occurred in two phases: a 2‐month period before the adoption of EAC at our health center and a 2‐month period after its implementation.

Patients were selected from the internal colonoscopy database, which included all patients who underwent colonoscopy during the study period. We excluded patients who underwent colonoscopy for specific colon‐related conditions or complications, such as melena, hematochezia, diarrhea, inflammatory bowel disease assessment, and a known history of colon cancer.

This study was approved by the Henry Ford Health Institutional Review Board on December 12, 2023 (IRB number 16810–01).

### 2.2 Study Cohort

Patients were stratified into two groups: those who had colonoscopy without EAC and those who had colonoscopy with EAC. Patients who had EAC were those who underwent colonoscopy with the Endocuff distal cap attachment, and all those who underwent conventional colonoscopy without Endocuff attachment were non‐exposed controls. Baseline demographic and clinical patient data were extracted, including age, sex, race, body mass index (BMI), comorbidities, previous anticoagulation therapy, and American Society of Anesthesiologists (ASA) grade. The following outcome and colonoscopy procedural characteristics were collected: bowel prep quality, estimated blood loss during the procedure, number of lesions detected (SSP and tubular adenomas), total procedure time, cecal intubations performed, cecal intubation time, and ileal intubations performed. All data were manually extracted from the electronic medical record. Racial categories were self‐reported in the medical record. Due to the predominance of patients identifying as Asian, Black, or White, the remaining racial categories were combined into an “other” group. Indications for colonoscopy were obtained from the medical record.

Demographic characteristics, colonoscopy‐related data, and outcomes were compared between the two groups. The primary outcomes were the detection rates of SSPs and tubular adenomas. Secondary outcomes included total procedure time, cecal intubation time, and ileal intubations performed. Bowel preparation quality was assessed qualitatively by the endoscopist at the time of the procedure, rather than using a specific scale.

### 2.3 Statistical Methodology

Continuous variables were described as mean ± standard deviation (SD), while categorical variables were presented as counts and frequencies. Group comparisons for continuous variables were performed using the two‐sample *t* test or the Wilcoxon rank‐sum test when the equality of variances assumption was not met. For categorical variables, the *χ* ^2^ test of independence was used, or the Fisher exact test if more than 20% of expected counts were less than 5.

Logistic regression analyses were employed, starting with univariate modeling and progressing to multivariable modeling. The multivariable model included gender, race, and quality of bowel preparation, which were selected a priori due to their known association with the primary outcome. The goal was to evaluate the association between the use of EAC and the detection of SSPs and tubular adenomas.

Statistical significance was defined as *p* < 0.05. All analyses were conducted using SAS 9.4 (SAS Institute, Cary, NC).

### 2.4 Baseline Characteristics of Patients Who Underwent Screening Colonoscopy

A total of 435 patients underwent screening colonoscopy during the study period, including 220 (51%) women and 215 (49%) men with a mean ± SD age of 57.6 ± 10.8 years. In the total group, 90 (21%) were Black, 277 (64%) were White, and 18 (4%) were in a different racial group. There were 173 patients (40%) who had BMI > 30 kg/m^2^, and most patients had not been on any previous anticoagulation therapy (97%) (Table 1).

**TABLE 1:** Baseline patient characteristics.

	All patients, *N* = 435	Conventional colonoscopy, *n* = 246	EAC, *n* = 189	*p*
Age, years	57.6 ± 10.8	58.1 ± 11.0	56.8 ± 10.4	0.20
Sex				0.02
Female	220 (51)	112 (46)	108 (57)	
Male	215 (49)	134 (54)	81 (43)	
Race				0.13
Asian	32 (7)	11 (4)	21 (11)	
Black	90 (21)	51 (21)	39 (21)	
Hispanic	2 (0)	2 (1)	0 (0)	
White	277 (64)	163 (66)	114 (60)	
Other	18 (4)	10 (4)	8 (4)	
Body mass index > 30 kg/m^2^	173 (40)	105 (43)	68 (36)	0.16
Comorbidities				
Coronary artery disease	15 (3)	11 (4)	4 (2)	0.18
Diabetes, type 2	60 (14)	39 (16)	21 (11)	0.16
Dyslipidemia	139 (32)	83 (34)	56 (30)	0.29
End‐stage renal disease	1 (0)	1 (0)	0 (0)	0.46
Hypertension	156 (36)	96 (39)	60 (32)	0.19
Myocardial infarction	2 (0)	2 (1)	0 (0)	0.21
Stroke	3 (1)	2 (1)	1 (1)	0.43
Previous anticoagulation therapy				0.48
None	424 (97)	238 (97)	186 (98)	
Clopidogrel/ticagrelor	3 (1)	2 (1)	1 (1)	
Direct oral anticoagulant	5 (1)	3 (1)	2 (1)	
Warfarin	3 (1)	3 (1)	0 (0)	
ASA grade				0.01
I	16 (4)	11 (4)	5 (3)	
II	380 (87)	205 (83)	175 (93)	
III	39 (9)	30 (12)	9 (5)	

*Note:* Data is shown as *n* (%) or mean ± standard deviation.

Abbreviations: ASA, American Society of Anesthesiologists; EAC, endocuff‐assisted colonoscopy.

### 2.5 Group and Procedural Characteristics

Among the 435 individuals who underwent screening colonoscopy, 246 (57%) had conventional colonoscopy and 189 (43%) underwent Endocuff‐assisted colonoscopy (EAC). The groups did not differ significantly in terms of sex, race, BMI, presence of comorbidities, or previous use of anticoagulation therapy. However, most patients were classified as ASA grade II (*n* = 380; 87%), and the ASA grade distribution was significantly different between the groups (*p* = 0.01). A larger proportion of patients in the EAC group were at ASA grade II compared to the conventional colonoscopy group (93% vs. 83%), while a smaller proportion were at ASA grade III (5% vs. 12%) (Table 1).

Most patients had satisfactory bowel preparation (95%), although the quality of bowel preparation differed slightly between groups (*p* < 0.01). A larger proportion of patients who underwent conventional colonoscopy had poor bowel prep quality (7% vs. 2%). Additionally, while no patients experienced blood loss of at least 100 mL, a slightly higher proportion of patients who underwent EAC had minimal blood loss (99% vs. 85%; *p* < 0.01) (Table 2).

**TABLE 2:** Procedural characteristics and clinical outcomes.

	All patients, *N* = 435	Conventional colonoscopy, *n* = 246	EAC, *n* = 189	*p*
*Procedural characteristics*				
Bowel prep quality				< 0.01
Satisfactory	415 (95)	229 (93)	186 (98)	
Poor	20 (5)	17 (7)	3 (2)	
Estimated blood loss				< 0.01
None	36 (8)	35 (14)	1 (1)	
Minimal	398 (91)	210 (85)	188 (99)	
100 mL	1 (0)	1 (0)	0 (0)	
Procedural outcomes				
Total procedure time, min	18.7 ± 7.5	19.0 ± 7.3	18.3 ± 7.7	0.36
Cecal intubation performed	431 (99)	245 (100)	186 (98)	0.20
Cecal intubation time, min	4.4 ± 3.3	4.5 ± 3.6	4.2 ± 2.8	0.31
Ileal intubation performed	160 (37)	134 (55)	26 (14)	< 0.01
*Main outcomes*				
Detected polyps per patient	1.7 ± 2.2	1.7 ± 2.2	1.9 ± 2.1	0.37
Sessile serrated polyps	51 (12)	21 (8.5)	30 (16)	< 0.01
Tubular/tubulovillous adenoma	175 (40)	97 (39)	78 (41)	0.70

*Note:* Data is shown as *n* (%) or mean ± standard deviation.

Abbreviation: EAC, endocuff‐assisted colonoscopy.

### 2.6 Main Outcomes

The mean total procedure time for all patients was 18.7 ± 7.5 min, with no significant difference between groups. Cecal intubation was performed in 99% of patients, with a mean cecal intubation time of 4.4 ± 3.3 min. A total of 160 ileal intubations were performed, with a significantly larger proportion of ileal intubations in the conventional colonoscopy group compared to the EAC group (55% vs. 14%; *p* < 0.01) (Table 2).

In total, 435 polyps were detected across both groups, with an average of 1.7 ± 2.2 polyps per patient. There were 51 SSPs and 175 tubular adenomas detected. While the overall mean number of polyps detected did not differ between groups, the EAC group had a significantly higher SSP detection rate compared to the conventional colonoscopy group (16% vs. 8.5%; *p* < 0.01). However, the adenoma detection rate was similar between the EAC and conventional colonoscopy groups (41% vs. 39%; *p* = 0.70) (Table 2). No procedural complications were reported in either group.

## 3 Discussion

In this cohort study of patients who underwent CRC screening colonoscopy with EAC versus conventional colonoscopy, we observed that both groups had very similar clinical and procedural characteristics; however, patients who underwent EAC had a significantly higher SSP detection rate, suggesting that the Endocuff colonoscope attachment may be useful for identifying these somewhat difficult‐to‐visualize lesions. Notably, the adenoma detection rate was similar for both approaches, emphasizing the non‐inferiority of EAC for this critical diagnostic capability.

Colonoscopy is widely accepted as the most effective method for CRC screening and prevention, although its effectiveness depends on the successful visual identification and removal of precancerous lesions. The adenoma detection rate is inversely related to the incidence of interval cancers, making it a standard metric for assessing colonoscopy quality, with a recommended minimum rate of 25% (30% for men and 20% for women) [9]. However, there is no established standard rate for the detection of serrated lesions, such as SSPs. SSPs are particularly challenging to identify due to their flat appearance against the colon wall, indistinct borders, and resemblance to benign lesions. This is concerning as SSPs are precursors in the serrated pathway to CRC, which progresses rapidly [10], accounting for 20% to 30% of all CRC cases [11].

CRCs that arise during the time between recommended colonoscopies are considered “interval cancers,” which are thought to be largely due to lesions that are missed during colonoscopy [10]. Importantly, SSPs are thought to account for a large portion of these interval cancers, particularly those of the proximal colon, due to their difficulty in detection and their ability to rapidly transition to dysplastic or invasive carcinomas [12]. One recent population‐based study found that an endoscopist's rate of proximal serrated polyp detection was inversely associated with interval cancer incidence, suggesting that tracking this rate in addition to adenoma detection rate could improve colonoscopy efficaciousness [13]. Thus, improved methods for accurately identifying SSP are needed.

In recent years, several new attachment devices, including the Endocuff, have been developed with the goal of improving colonoscopy visualization and lesion detection. To better understand the utility of these devices in improving the effectiveness of screening colonoscopies, understanding how such devices impact the detection rates of both tubular adenomas and SSPs is essential. In our study, SSPs were identified at a higher rate with EAC, without negatively affecting other procedural factors such as time and complication rates. Although limited data are available regarding SSP detection rates with EAC compared to the extensive data on ADR, recent meta‐analyses have indicated significantly higher SSP detection rates with EAC [14, 15, 16, 17]. However, one meta‐analysis that assessed several different projection‐containing attachment devices (Endocuff, Endocuff Vision, Endo Rings, and Wing Cap) reported a higher detection rate of adenomatous polyp detection for Endocuff, but no significant differences in sessile polyp detection [18]. Interestingly, while many studies have suggested that Endocuff use increases the detection of adenomatous and total polyp detection, we did not observe significant differences in these parameters [15, 17, 18, 19].

Our study demonstrated no significant differences in various procedural quality metrics between EAC and conventional colonoscopy, including cecal intubation rates, cecal intubation time, and total procedure time, which is consistent with other reports [15, 17, 19]. However, patients undergoing EAC were less likely to have had ileal intubation performed, although it is important to note that ileal intubation was not attempted in many cases as the indication for these exams was routine CRC screening or surveillance. Few studies have addressed ileal intubation rates in patients undergoing EAC. One randomized controlled multicenter trial of 500 patients in Germany reported a significant increase in sessile serrated lesion detection with EAC but found no significant difference in ileal intubation rates [20]. Another randomized controlled trial of 337 patients reported a significantly higher adenoma detection rate and decreased ileal intubation rate with EAC [21]. Many consider ileal intubation to be a marker for a complete colonoscopy; however, the clinical utility of this maneuver has been called into question, especially in patients undergoing colonoscopy for routine CRC screening. Studies have shown that ileal intubation has limited utility in asymptomatic patients, but it may be useful in detecting abnormalities in symptomatic patients, particularly those presenting with right lower quadrant abdominal pain or diarrhea [22, 23]. Our data indicate that EAC may limit successful ileal intubation, but in asymptomatic patients undergoing routine screening and surveillance colonoscopies, ileal intubation may not be necessary to perform.

Evaluating the potential impact of EAC on patient safety and experience is also crucial. Although there was no significant difference in anticoagulation or antiplatelet use between the two groups in our study, patients who underwent EAC were slightly more likely to experience minimal blood loss than those in the traditional colonoscopy group. However, no significant blood loss or injury was reported in either group. While some studies have reported an increased risk of minor complications with EAC, most commonly superficial mucosal injuries [17, 18, 24, 25], others have noted challenges with Endocuff device removal, leading to device detachment or the need for Endocuff removal due to patient discomfort or severe diverticulosis [15, 26]. The increased incidence of minimal bleeding in our EAC group is consistent with findings that this device may cause some additional mild mucosal trauma compared to traditional colonoscopy, although this does not appear to be clinically relevant. Importantly, there were no procedural complications in either group. Future studies focusing on patient experience and adverse events would be useful to provide additional insight into how EAC may affect patient comfort.

One limitation of this study, as with other studies assessing EAC, is the inability to blind endoscopists. The awareness of using a novel device alone could increase the diligence of operators, potentially leading to higher polyp detection rates. Additionally, about 7% of patients in the conventional colonoscopy group had poor bowel preparation compared to only 2% in the EAC group, which may have impaired SSP detection in some patients. This study also focused specifically on patients undergoing screening and surveillance colonoscopies, excluding those presenting with other gastrointestinal symptoms. Moreover, we were unable to assess the performance of EAC in patients with challenging anatomical characteristics, such as a tortuous or redundant colon. Further research focusing on EAC performance across various anatomical factors would be valuable in identifying the optimal patient population for this technique.

Overall, the challenge and importance of detecting SSPs through colonoscopy are increasingly being acknowledged. Our study suggests that EAC can enhance the detection of these lesions without posing significant risks to patients or hindering other procedural metrics such as cecal intubation rate and total procedure time. However, clinicians should be aware that EAC might limit the ability to perform ileal intubation and may not be ideal for patients with specific ileal pathology. Although our study did not find any significant benefits of EAC for tubular adenoma and total polyp detection, the improved SSP detection with EAC highlights the potential of this device, or similar attachments, to enhance the identification of hard‐to‐visualize lesions. This, in turn, could help reduce the incidence of interval cancers.

## Conflicts of Interest

The authors declare no conflicts of interest.
